# A Benchmark of Parametric Methods for Horizontal Transfers Detection

**DOI:** 10.1371/journal.pone.0009989

**Published:** 2010-04-01

**Authors:** Jennifer Becq, Cécile Churlaud, Patrick Deschavanne

**Affiliations:** 1 Dynamique des Structures et Interactions des Macromolécules Biologiques, Institut National de la Santé et de la Recherche Médicale (INSERM) UMR-S 665, Université Paris Diderot, Institut National de la Transfusion Sanguine, Paris, France; 2 Molécules Thérapeutiques in silico, Institut National de la Santé et de la Recherche Médicale (INSERM) UMR-S 973, Université Paris Diderot, Paris, France; Institut de Pharmacologie et de Biologie Structurale, France

## Abstract

Horizontal gene transfer (HGT) has appeared to be of importance for prokaryotic species evolution. As a consequence numerous parametric methods, using only the information embedded in the genomes, have been designed to detect HGTs. Numerous reports of incongruencies in results of the different methods applied to the same genomes were published. The use of artificial genomes in which all HGT parameters are controlled allows testing different methods in the same conditions. The results of this benchmark concerning 16 representative parametric methods showed a great variety of efficiencies. Some methods work very poorly whatever the type of HGTs and some depend on the conditions or on the metrics used. The best methods in terms of total errors were those using tetranucleotides as criterion for the window methods or those using codon usage for gene based methods and the Kullback-Leibler divergence metric. Window methods are very sensitive but less specific and detect badly lone isolated gene. On the other hand gene based methods are often very specific but lack of sensitivity. We propose using two methods in combination to get the best of each category, a gene based one for specificity and a window based one for sensitivity.

## Introduction

Horizontal gene transfer (HGT) between unrelated species is thought to be one of the leading creative forces driving bacterial evolution [1], [2], [3], [4], [5], [6], [7]. If a horizontally acquired gene is to be kept and expanded within a bacterial population, it must confer a selective advantage upon the host species and increase its fitness for instance for colonizing new environments or new hosts [6], [8], [9], [10]. HGTs are known to be of a great importance in virulence or antibiotic resistance acquisition by prokaryotes [8], [11], [12], [13]. Therefore to understand the evolution of a prokaryote, it is crucial when one is analyzing a newly sequenced genome to distinguish the species-specific regions from the horizontally acquired ones.

For exhaustive determination of horizontal transfers in a given genome, methods based on phylogenetic incongruencies are not well suited. Indeed, for this kind of methods, a correct number of orthologs for each gene is needed to produce a phylogenetic tree that could be compared to the species tree. Even with their ever increasing sizes, genomic databases are still lacking orthologs for in general over 50% of a newly sequenced genome [14], [15], [16], [17].

On the other hand, so called parametric methods, that is those based on the compositional characteristics, such as GC content, codon usage and di- and tetra-nucleotide frequencies of a genome are well suited to determine exhaustively all the horizontal transfers of a genome. These usually easily applicable methods require only the genome of the organism under study. They are based on the fact that the genomic compositional characteristics are specific to each species [18], [19], [20], [21], [22], [23]. Therefore, by studying the compositional fluctuations along a genome, one can extract atypical genes/fragments that are potentially from exogenous origin because they present compositional characteristics different from the majority of the studied genome.

However, more than two dozens parametric methods have been developed since 1991 (for instance [24], [25], [26], [27], [28], [29], [30], [31], [32], [33], [34], [35], [36], [37], [38], [39]), and furthermore it has been shown that these different methods don't always extract the same fragments and even are sometimes contradictory [26], [40]. For most of these methods, they were developed and directly applied to prokaryotic genomes without questioning their efficiency. Rarely would some authors introduce exogenous genes into a given genome and assess the ability of their newly developed method to detect, among the real horizontal transfers already present in the genome, these artificially introduced genes [37], [41]. This methodology still presents some inconvenience because it allows one to evaluate a method only in terms of sensitivity – the ability to detect all horizontal transfers – and not in terms of specificity – the ability to avoid detecting native genes – because by definition, the horizontal transfers already present in the genome under study impede this evaluation.

Azad and Lawrence have developed artificial genomes modeled from real genomes from which every atypical region was removed [42]. By combining these genomes one can create a whole panel of model genomes containing a known content of horizontal transfers in order to assess the efficiency of parametric methods in ideal conditions, and most of all according to different conditions. Indeed, it has been suggested that if these methods detect different fragments it is because there are different types of horizontal transfers more or less ameliorated [43] as a function of the time elapsed in the genome [40], [44].

In this paper, we present the comparative analysis of 16 parametric methods in order to assess their ability to detect horizontal transfers in model genomes according to (i) their species of origin, (ii) their overall quantity in the host genome and (iii) their mean size in terms of number of genes.

## Materials and Methods

### Sequences

Azad and Lawrence kindly provided us with 11 artificial genomes modeled from: *A. fulgidus* DSM 4304, *B. subtilis* 168, *D. radiodurans* R1 chromosome I, *E. coli* K12, *H. influenzae* Rd KW20, *M. jannaschii* DSM2661, *N. gonorrhoeae* FA1090, *R. solanacearum* GMI1000, *S. meliloti* 1021, *Synechocystis* sp. PCC6803 and *T. maritime* MSB8 [42]. These genomes were used to create model genomes in which the position and origin of each horizontal transfer is known. For all the model genomes, *E. coli* was the host genome – receiving the horizontal transfers – and the 10 other genomes were the source of these transfers. In the “recipient genome analysis”, the host genome in swapped between 7 of the artificial genomes.

These genomes were ranked according to their distance in terms of tetranucleotidic frequencies (*a.k.a* the genomic signature) to the *E. coli* genome (Table 1) and three groups were defined: *“close”* (represented in shades of blue in Supplementary Figure S1), *“intermediary”* (in shades of green) and *“far”* (in orange, red and pink) genomes. Due to the artificial characteristics of the model genomes (42), this ranking is operational and cannot be compared to the corresponding species phylogenetic relationships.

**Table 1:** Classification of the 6 gamma proteobacteria and the artificial genomes used in this study according to their distance to artificial *E. coli*.

Species	GC%	Group	Distance (AU)*	Code	Color
***Escherichia coli***	50.8	Gamma-Proteobacteria	-	Ecol	Grey
***Salmonella enterica***	51.9	Gamma-Proteobacteria	84	Sent	Light green
***Erwinia pyrifoliae***	53	Gamma-Proteobacteria	103	Epyr	Light blue
***Serratia proteomaculans***	55	Gamma-Proteobacteria	132	Spro	Green
***Yersinia pestis***	47.7	Gamma-Proteobacteria	152	Ypes	Red
***Vibrio cholerae***	47.5	Gamma-Proteobacteria	192	Vcho	Light red
***Klebsiella pneumoniae***	56.9	Gamma-Proteobacteria	194	Kpne	Blue
***Neisseria gonorrhoeae***	52.7	Beta-Proteobacteria	247	Ngon	Light blue
***Bacillus subtilis***	43.5	Firmicute	274	Bsub	Dark blue
***Synechocystis sp.***	47.4	Cyanobacteria	294	Ssyn	Cyan
***Archaeoglobus fulgidus***	48.1	Archaea	332	Aful	Dark green
***Haemophilus influenzae***	38.1	Gamma-Proteobacteria	385	Hinf	Light green
***Sinorhizobium meliloti***	62.2	Alpha-Proteobacteria	397	Smel	Green
***Thermotoga maritima***	46.2	Thermotogale	402	Tmar	Pink
***Deinococcus radiodurans***	67.0	Deinococci	463	Drad	Brown
***Ralstonia solanacearum***	67.0	Beta-Proteobacteria	486	Rsol	Fuchsia
***Methanocaldococcus jannaschii***	31.3	Archaea	618	Mjan	Orange

*Distances are calculated using Euclidian metric between the frequencies of the 256 tetranucleotides of each genome. The color-code correspond to the one used in Supplementary Figure S1.

To assess the performance of the methods in detecting HTs originating from very close species that was not possible with the artificial genomes, we used 6 gamma-proteobacteria as source genomes for HTs: *Erwinia pyrifoliae* Ep1/96, *Klebsiella pneumoniae* 342, *Salmonella enterica* subsp. enterica serovar Typhi CT18, *Serratia proteamaculans* 568, *Vibrio cholerae* O395 chromosome I and *Yersinia pestis* KIM10.

### Methods tested

Two types of parametric methods exist: those based on a scoring metric to evaluate the atypicity of a fragment [26], [27], [28], [29], [30], [34], [36], [37], [38], [39], [45] and those based on classifications to separate native genes from atypical genes [24], [25], [31], [33], [35]. The first will consider as horizontal gene transfers those presenting a score higher (or lower) than a certain threshold. The second usually define the atypical groups according to (i) the function of the genes in each group [25], [33] or to (ii) the mean score of the group, which depends on a scoring metric and external information or some known characteristics of the genome under consideration [24], [31], [35]. As the artificial genomes contain no annotation, only methods based on a metric scoring system were evaluated.

Sixteen different methods were tested, that are the most representative of the different criteria evaluated – GC content, codon usage, amino-acid usage, dinucleotide and tetranucleotide frequencies – and of the different metric measures (Table 2). The eight different metrics used by the methods are recalled in Supplementary Table S1.

**Table 2:** The sixteen horizontal transfer detection methods analyzed in this paper.

Name	References	Criteria	Genome scanning	metric
GC.windows	[27], [29], [38]	GC%	20 kb windows, 5 kb step	Manhattan
GCtotal	[27]	GC%	Genes	None
GC1-GC3	[27], [30]	GC% in positions 1 and 3 of genes	Genes	None
dint5	[29], [38], [39]	Normalized dinucleotides	5 kb windows, 5 kb step	Delta*
dint.di31T2	[28]	Normalized dinuleotides in position 3∶1 of codons	Genes	Mahalanobis
CU.chi2	[30]	Codons	Genes	Chi2
CU.karlin	[29], [38]	Codons	Genes	Delta*
CU.karlin.aa	[29]	Amino acids	Genes	Delta*
CU.KL	[31]	Codons	Genes	Kullback-Leibler
CU.mahalanobis	[27]	Codons	Genes	Mahalanobis
oli.Pearson	[37]	Normalized tetranucleotides	5 kb windows, 1 kb step	Correlation
oli.covariance	“	Normalized tetranucleotides	5 kb windows, 1 kb step	Covariance
oli.chi2	“	Normalized tetranucleotides	5 kb windows, 1 kb step	Chi2
oli.mahalanobis	“	Normalized tetranucleotides	5 kb windows, 1 kb step	Mahalanobis
oli.KL	“	Normalized tetranucleotides	5 kb windows, 1 kb step	Kullback-Leibler
signature	[26]	Tetranucleotides	5 kb windows, 0.5 kb step	Euclidian

Usually, the value of a given criteria for the complete genome either correspond to the mean value of the criteria calculated over all the genes or to the value calculated over the whole genome. The signature method is quite different for this point because the mean tetranucleotide frequencies for the whole genome are not calculated over all the windows but over the majority of windows from which were removed, after classification, windows that were too atypical [26].

### Threshold evaluation

In order to evaluate the efficiency of each method, we tried to use them as described in the literature. However, the determination of the correct threshold is usually a critical issue as it usually depends on the genome under consideration. Thus, in order to be able to compare the methods in the same conditions, we established a common protocol to determine an operational threshold giving the best results in terms of errors for each method.

For this issue, we used “standard” model genomes containing 9% of horizontal transfers (a median value taking into account the published % of HGTs for numerous genomes [46]) originating in equivalent proportions from *B. subtilis*, *N. gonorrhoeae*, *S. meliloti*, *A. fulgidus*, *M.jannaschii* and *R. solanacearum*, *i.e*. 2 “close”, 2 “intermediary” and 2 “far” genomes. The sizes of the horizontal transfers were comprised between 1 and 15 genes. As certain parameters are random (the distribution of the transfers in the genome, the sizes, etc.) we generated 5 “standard” model genomes in these conditions.

For each of these 5 genomes, we calculate a score per gene or per window according to each method. To allow comparisons between methods using sliding windows and using gene based calculations, we reported each score per window into scores per genes. The score of each gene corresponds to the barycenter of the scores of the windows containing the gene weighted by the size of the portion of the gene in each window. We then realized boxplots with the scores of each method to establish the atypicality thresholds without needing to assume an underlying statistical distribution of these scores. Any data observation that lies beyond the extremities of the whiskers of a boxplot is considered as an outlier. These extremities *S*_inf_ and *S*_sup_ are calculated as following:
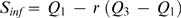


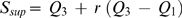
where *Q_1_* and *Q_3_* are the first and third quartile of a distribution, and (*Q_3_* − *Q_1_*) the interquartile distance. The whiskers extension is proportional to a given factor *r*. Because we know which genes should be detected as HGT and those that shouldn't we could realize ROC-like curves for each method by varying the value of this whiskers extension factor r (and therefore the threshold values) from 0.5 to 4 by 0.5 steps. Subsequently we established the optimal value of *r* for each method as the one that minimizes the mean error in terms of sensitivity and specificity. The mean error is calculated as following:
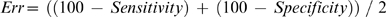



We considered as stable the methods for which the standard deviation of the optimal *r* over the 5 genomes was below 0.5, *i.e.* the value of the step for *r*.

## Results

### Comparison and efficiency of the methods over the “standard” model genomes

**Figure 1:**
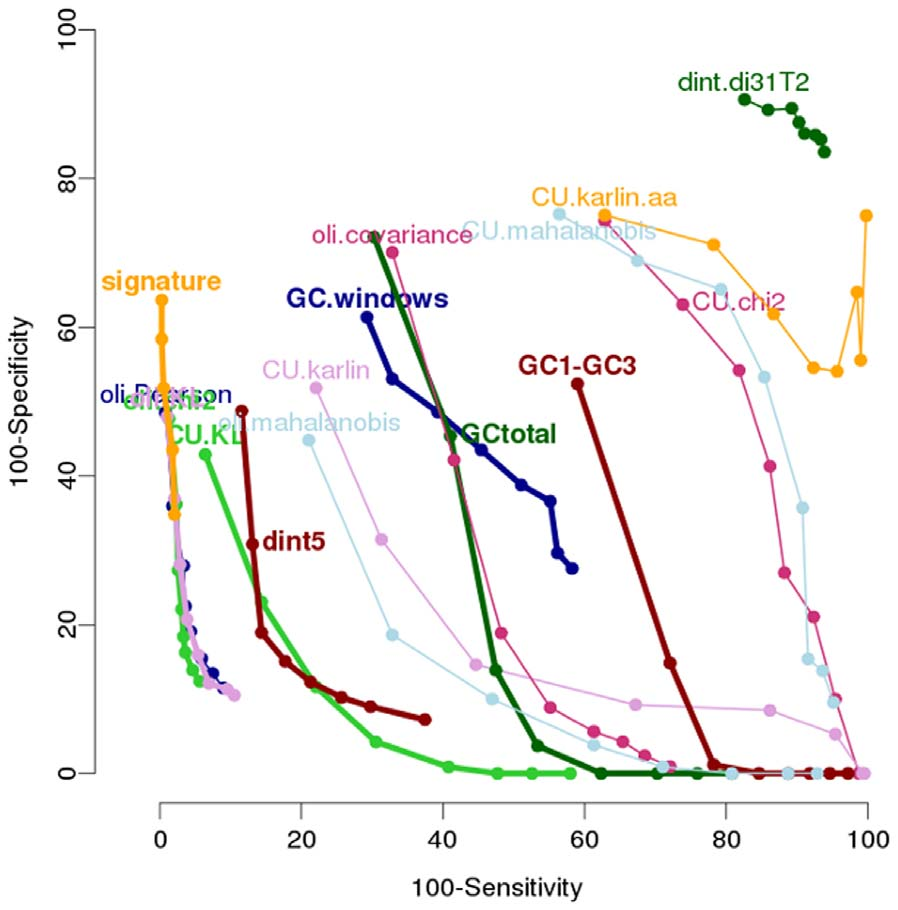
ROC-like curves of the 16 methods. Each dot of a curve corresponds to the values of type I error (100-sensitivity) and type II error (100-specificity) for each value of *r* (see M&M). The best methods are those with the less errors, *i.e.* those that are the closest of the origin.

The performance of each method over 5 “standard” model genomes is shown in Supplementary Figure S1, recapitulated in Table 3 and their comparison is presented in the ROC-like curves in Figure 1. Even in these ideal conditions for the utilization of the methods – because each host genome is more homogenous than natural ones and none of the artificially introduced genes has been ameliorated – not all the methods present the same efficiency (Figure 1). Indeed, the methods are uniformly distributed over the ROC-like curve graphic. Some are particularly inadequate in our conditions (dint.di31T2 for example, in the top right of the graphic) whereas others are very effective (oli.chi2 for example, near the origin).

**Table 3:** Mean performances of all the 16 methods with “standard” model genomes.

Methods	Sensitivity	Specificity	Threshold ± *deviation* *
GC.windows	56.6	51.6	1.8±*1.4*
GCtotal	49.1	96.1	1.9±.*2*
GC1-GC3	23.9	98.2	1.4±.*2*
Dint5	79.4	84.4	1.8±.*3*
Dint.di31T2	16.8	9.5	0.5±.*0*
CU.chi2	1.3	100	4.0±.*0*
CU.karlin	62.2	73.4	1.1±.*2*
CU.karlin.aa	65.9	26	0.5±*0*
CU.KL	77.2	87.8	1.4±.*2*
CU.mahalanobis	3.9	79.8	3.6±.*5*
oli.Pearson	92.5	85.5	3.2±.*8*
oli.covariance	38.8	91.5	2.2±.*4*
oli.chi2	93.8	87.1	3.9±.*2*
oli.mahalanobis	64.9	81.6	1.1±.*2*
oli.KL	91.5	89.2	3.6±*0.4*
signature	98	67.3	1.5±.*0*

*Threshold corresponds to the value of r *(see M&M) for optimal performance; the standard deviation of optimal* r *over the 5 tested genomes is precised*.

Moreover, this comparative analysis allows us to point out the weaknesses of each method (if any):

- Some compositional criteria don't distinguish atypical regions from the native genes. This is the case for dinucleotides in position 3∶1 of genes (dint.di31T2) and for amino-acid usage (CU.karlin.aa)
- Some metric measures are not adequate for separating atypical fragments from native ones. This is true for chi2 metric used with codon usage (CU.chi2), covariance used with tetranucleotide frequencies (oli.covariance) and the Mahalanobis distance. Regardless of the criteria used (codon usage “CU”, dinucleotide frequency “dint” or tetranucleotide frequency “oli”), it appears that the methods using Mahalanobis distances as the metric measure (dint.di31T2, CU.mahalanobis, oli.mahalanobis) always present lesser sensitivity than the other methods using the same criteria with another metric.
- Some methods such as GCtotal, GC1-GC3, CU.karlin are very sensitive to the origin of horizontal transfers. In these cases, it appears that fragments originating from close genomes (see M&M) present scores similar to those of the native genes (This is illustrated in Supplementary Figure S1). This point was further investigated by measuring the performances of each method according to the origin of artificial horizontal transfers (see below).

After such a comparative analysis, we reduced our set of methods to those that were the most effective for each of the four criteria, that is: GCtotal, GC1-GC3, dint5, CU.KL, oli.chi2, oli.KL and signature.

### The influence of horizontal transfer (HT) characteristics

#### Influence of HT origin

**Figure 2:**
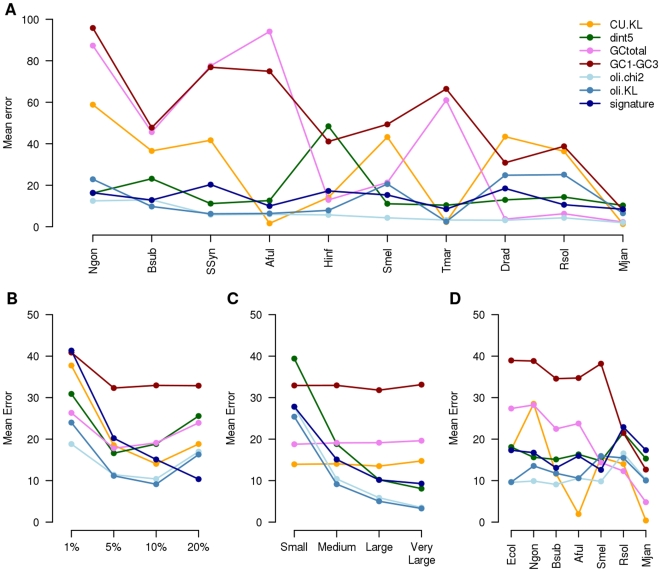
Mean errors of 7 methods according to (A) origin, (B) overall quantity, (C) size and (D) recipient genome. The mean error is the mean of type I (sensitivity) and type II (specificity) errors. It is presented here for the 7 efficient HT detection methods of each criterion (codon usage: CU.KL; dinucleotide frequencies: dint5; GC content: GCtotal and GC1-GC3; and tetranucleotide frequencies: oli.chi2, oli.KL and signature) according to four parameters. A: the origin. The unique donor genome of the HTs are ordered according to their distance to the host genome (*E. coli*) in terms of tetranucleotide frequencies – the closest on the left and the farthest on the right. B: the overall quantity of HTs in percentage of the genome. C: the size of the HTs. Small, Medium, Large and Very Large respectively mean 1 to 5 genes, 5 to 10 genes, 10 to 20 genes and 20 to 30 genes. D: the host genome, *i.e.* the genome receiving the HTs.

Ten types of model genomes were realized in which the source of HTs is unique. In each of these genomes, there are 12.5% of HTs from 5 to 10 genes long originating from only one of the 10 donor species. The detection mean error of the 7 tested methods is presented in Figure 2A. On the x-axis of this figure, the HT source genomes are ordered according to their distance to the model genome of *E. coli* in terms of signature (as presented in Table 1), the closest on the left and the furthest on the right. The tetranucleotide based methods (oli.chi2, oli.KL and signature) present a very good efficiency (mean error <20%) regardless of the HT genome origin. The dinucleotide-based method (dint5) is almost as powerful, being sensitive to only one source out of 10 *(H. influenzae)*. At last, the mean errors of the gene based methods (GCtotal, GC1-GC3 and CU.KL) are usually quite high – mean errors are respectively 41%, 53% and 28% – and vary considerably – mean standard deviations are respectively 37%, 26% and 21% – according to HT origin. By comparing the GC contents of the *E. coli* model genome and the HT source model genomes for which these methods are the less efficient, *N. gonorrhoeae*, *Synechoccystis sp.*, *A. fulgidus* and *T. maritima*, it appears that these model genomes are those presenting almost the same GC% as *E. coli*. GC content and codon usage criteria are not discriminant enough to distinguish foreign DNA from native DNA in a given genome compared to di- or moreover tetranucleotides.

To investigate further the heterogeneity of performance of the methods according to HT origin, model genomes were created by integrating to the artificial *E. coli* genome 9% of HTs originating in equivalent proportion (1.5%) from the 6 real gamma-proteobacteria genomes mentioned in the Materials and Methods section. Because these genomes are phylogenetically very close to *E. coli*, this allows us to assess the performance of the methods in very difficult conditions. Sensitivity, specificity and mean error are presented in Table 4. It appears that only two tetranucleotide based methods (oli.chi2 and signature) present satisfying results – less than 30% of errors – for HT detection in these conditions.

**Table 4:** Sensitivity, specificity and mean performance of the methods with HTs originating from real gamma-proteobacteria.

Method	Sensitivity	Specificity	Mean error
GCtotal	5.32	100	47.34
GC1-GC3	2.64	100	48.68
CU.KL	6.19	96.95	48.43
dint5	39.66	77.73	41.3
oli.chi2	72.82	84.21	21.48
oli.KL	61.01	70.12	34.44
signature	84.82	59.23	27.97

#### Influence of HT quantity

Four types of model genomes were generated containing respectively 1%, 5%, 10% and 20% of HTs. In these genomes, the HTs emanated from three donor species, a close one *B. subtilis*, an intermediate one *S. meliloti* and a far one *M. janaschii*. These donor species were chosen because there are the HT donors of each distance category (close, intermediate and far) for which the methods are the most performing according to the previous analysis. The sizes of the HTs were comprised between 5 and 10 genes. The mean error of the 7 methods according to overall HT quantity is presented in Figure 2B. For all the methods but one (signature), the mean error curves are in a open U shape, meaning that the scoring discrimination is sensitive to too low (1%) or too great quantities (20%) of HTs, but is adequate to mean quantities of HTs (5 to 10%). The signature method is the less efficient method when there are too few HTs but it gets more and more efficient as HT quantity increases, even for 20%. This particularity is the direct consequence of calculating the mean host genome signature over only a subset of the genome, by excluding atypical fragments after classification (see M&M). It is to be noted that the GC1-GC3 method is quite inefficient whatever the quantity of HTs (Figure 2B).

#### Influence of HT size

Four types of model genomes were realized in which the average size of the HTs was small (1 to 5 genes), medium (4 to 10 genes), large (10 to 20 genes) or very large (20 to 30 genes). For the reasons presented above, the donor species were *B. subtilis*, *S. meliloti* and *M. janaschii*. The overall quantity of HTs in these genomes was fixed to 10%, as it appeared to be the optimal quantity for the majority of the methods. The mean error of the 7 methods according to HT size is presented in Figure 2C. Two types of curves can be distinguished: flat ones – mean error is constant regardless of the size of the HTs – corresponding to the gene based methods (GCtotal, GC1-GC3 and CU.KL) and decreasing ones – mean error decreases as HT size increases – corresponding to the window based methods. As these methods scan the genome by using 5 kb windows, they are – for once – less able to detect small HTs compared to the gene based methods.

#### Influence of host genome

Seven types of model genomes were realized for which the host genome, *i.e.* the one receiving the HTs, is different each time. The seven host genomes are artificial *E. coli*, *N. gonorrhoeae*, *B. subtilis*, *A. fulgidus*, *S. meliloti*, *R. solanacearum* and *M. janaschii*. For each of these host genomes there are 9% of HTs originating in equivalent proportion (1.5%) from the 6 other genomes. The mean error of the 7 methods according to the host genome is presented in Figure 2D. As for the influence of HT source, tetranucleotide-based methods (oli.chi2, oli.KL and signature) and the dinucleotide-based method (dint5) are less sensitive to host variation compared to GC based methods (GCtotal and GC1-GC3) or codon usage based methods.

### Combination of methods

The previous analyses show that the tetranucleotide based methods are the most adequate methods to use to detect HTs in most cases. But, because they cannot detect small HT – *i.e.* less than 5 genes long – we decided to combine the use of a tetranucleotide based method with the best gene based method the CU.KL method using the codon usage criteria with a Kullback-Leibler metric.

#### Over a “standard” model genome

We applied the methods on the 5 “standard” model genomes described above and detected as HTs those that were atypical for at least one method, *i.e.* we used the union of the detections. Mean sensitivity, specificity and errors are presented in Table 4 for each pair of methods. Compared to the use of one method alone, the combination improves sensitivity: 93.8% to 97.0% for oli.chi2, 91.5% to 96.2% for oli-KL and 98% to 99.4% for signature, but on the other hand specificity worsens. Because uniting the results of 2 methods overall raises the number of detected regions, it increases the number of true positives (increases sensitivity) and the number of false positives (decreases specificity) (Figure 1). The method combination presenting the less errors is CU.KL with oli.KL, however it is not very different from the other combinations.

#### Over a real genome

In the previous analyses, we applied the methods on artificial genomes that were developed by Azad and Lawrence [42]. The sequences of these genomes may present too little intrinsic variability compared to a real genome and therefore bias the performances of the HT detection methods. Thus, we decided to evaluate the performance of the methods combination by using a real genome, that of *E. coli* K-12 sp. MG1655. But as mentioned previously, a real genome already has its own HTs that could interfere with the specificity measurements, as they would likely be detected by the methods. We have therefore decided to remove from the *E. coli* genome all the genes detected by at least one of all the published methods used on this species. Out of the 4252 genes, 1592 were detected by one of the 6 methods referenced by Dufraigne *et al.* [26]; all of these genes were taken out of the genome. On the other hand, all non-coding regions perhaps presenting compositional divergences were maintained, *i.e.* the intrinsic variability of the genome is taken into account, unlike in the artificial genomes used previously. We then added to this “reduced” genome 9% of HTs from 1 to 15 genes originating from the same 6 artificial genomes used to generate the “standard” model genomes (see M&M). However, as noted previously the artificial genes inserted in the *E. coli* genome were not ameliorated as in the model genomes. This protocol was iterated 5 times to generate 5 “real” *E. coli* genomes over which we could evaluate the performance of the combinations of methods. The values of sensitivity, specificity and mean error are presented in Table 4 for each of the 6 pairs of methods. It appears that the sensitivity of the methods is slightly reduced compared to the use of standard genomes, whereas the specificity remains constant. The decrease in sensitivity is clearly due to the increasing intrinsic variability of the reconstructed *E. coli* genome. The fact that the specificity does not change indicates simply that, even if the threshold is increased, all the peaks above it are representative of HTs.

## Discussion

The artificial genomes kindly provided by Azad and Lawrence present “ideal” conditions to test horizontal transfer (HT) detection methods based on nucleotidic composition; first, due to their construction, they present low intrinsic variability and second the HTs introduced in these genomes are not ameliorated, *i.e.* they haven't started to gain the host genome compositional characteristics. They allowed us to realize an exhaustive evaluation of the different types of score based parametric methods used for horizontal transfer detection. All compositional criteria – GC%, codon and amino-acid usage, di- and tetranucleotide frequencies (Table 2) – were represented, using a wide range of metric distances – Manhattan distance, Euclidean distance, covariance measure, correlation measure, chi2 metric, Mahalanobis distance and Kullback-Leibler divergence (Supplementary Table S1). By applying these 16 methods in the same conditions, *i.e.* on the same genomes, with the same best threshold estimation process, we were able to compare the performance of the methods we tested in terms of both sensitivity and specificity. These conditions are not real ones, and the most efficient methods could perhaps not be the best in real situations, in particular if different methods detect different classes of HTs [40], [47], [48]. However this is not true for inefficient methods, if they perform badly in ideal conditions it is not likely they would perform better in real conditions.

Our first conclusion is that not all methods are suited for HT detection (Figure 1). It appears that the methods present a very variable efficiency, from those quite unable to detect, in our conditions, some HTs to that which are able to detect almost all (Figure 1). This great diversity in efficiency is quite amazing and was not predictable. Indeed some criteria are unable to distinguish foreign DNA (dinucleotides in position 3∶1 for example), and some metrics cannot separate the HTs from the native genes (Mahalanobis distance for example). Also the combination of a criterion and a metric can be critical. For example, using codon usage as criterion, some metrics are not suitable (chi2 metric and Mahalanobis distance) when some work well (Karlin delta* metric and Kullback-Leibler distance) (Figure 1 and Table 3). Base composition was already shown to be a weak indicator of horizontal transfers due to a number of biases in normal conditions and it is verified in this study as in the best conditions the mean error is a bit less than 30% (Figure 1 and Table 3) [49], [50], [51]. As a rule, tetranucleotide usage is a better indicator for horizontal transfer detection. Once again the metric used to analyze such criterion is essential, nevertheless sensitivity to the metric is less than with the other criteria as only the covariance metric and the Mahalanobis distance lead to poor results with tetranucleotides.

We pursued the analysis further by evaluating the performance of each method according to the different characteristics of the horizontal transfers. The four characteristics used in this analysis were the origin, the overall quantity, the size of the HTs and the recipient genome (Figure 2).

There is a great variability in efficiency for the methods tested as a function of the origin of the HTs (Figure 2A). It appears that the GC content, codon usage and dinucleotide based methods are far more sensitive to HT origin than those using tetranucleotides. The three former criteria can coincidentally be similar between a host and a donor genome, even if those are phylogenetically distant, and it has been noted that these criteria were not discriminative enough for HT detection [49], [50], [51]. On the other hand, it was shown that tetranucleotide frequencies are species-specific [20], [52], and therefore are more suited to distinguish foreign DNA in a given genome. However, when HTs originate from very closely related species, it is to be noted that even tetranucleotide-based methods perform less well than with “farther” HTs (Table 4) while still more efficient that the others in these difficult conditions.

HT quantity is also a parameter to take into account as in general this parameter influences the threshold determination (Figure 2B and Table 3). With the exception of GC1-GC3 which responds poorly whatever the global quantity of HT and the signature method which improves when the quantity increases, all the other methods present the same type of behavior with a maximum efficiency between 5 and 10% of HTs, range which is in general reported in the literature [26], [46].

The HT size parameter is the one that discriminates best between the two main types of criteria. Indeed, gene based criteria are independent of the HT size while the window-based oligonucleotide methods are very sensitive to this parameter (Figure 2C). Window based methods due to their processing mode are disadvantaged for small sizes of HTs and begin to be efficient only for medium sizes. This result is consistent with what is expected of such methods and is of interest when using a combination of method (see below). The best gene based method is CU-KL that overcomes methods based on base composition of codons. Again tetranucleotide window based methods are slightly more efficient that dinuleotide ones.

A change in recipient genome reveals the robustness of the methods in varying conditions (Figure 2D). Base composition based methods are very sensitive to a change in recipient genome. This might be imputable to the intrinsic variability of the recipient genome even in our conditions where this variability is reduced. The codon usage based method also exhibits a great variability in efficiency that is unexpected due to the variety of gene characteristics inserted in the genomes. As previously oligonucleotide-based methods are the most robust when the recipient genome changes, they present a weak dependence to the recipient genome.

Thus, two conclusions can be drawn here: first the “different methods for different HTs” statement is mainly due to the origin of the HTs, and second even though it seems to be true for GC content, codon usage or dinucleotide based methods, it doesn't apply to tetranucleotide based methods which look rather insensible to all HT criteria. Indeed, whatever the conditions tested here the oligonucleotide-based methods are the most performing in all conditions. This type of method works best even in difficult condition as it was the case for detecting HT originating from closely related species (Table 4) or when using a ‘real’ *E. coli* recipient genome (Table 5). However as tetranucleotide frequencies can only be computed over large sequence fragments to avoid statistical bias, small HTs (less than 5 genes long) will not be detected by these methods (Figure 2C). This could be inconvenient if the introduction of a long stretch of foreign DNA in a genomic sequence is followed by an important subsequent gene loss of this fragment by selective pressure, leaving only a few genes left, hard to detect by the tetranucleotide based methods. Therefore, we suggest to combine such a method with a gene based method to improve the sensitivity of detection. As it appears along this study, among the gene based methods, codon usage is the most discriminative criterion combined with a Kullback-Leibler measurement, and thus CU-KL is the gene-based method the most efficient for HT detection. It is still recommended to investigate further a gene presenting an atypical codon usage as it could be due to other causes, such as over-expression, bias amino-acid composition or repetition for instance [53].

**Table 5:** Mean performance of the combination of 2 methods over the “standard” model genomes and over the “real” *E. coli* genomes.

	Method combination	Sensitivity	Specificity	Mean error
**Standard genomes**	CU.KL – oli.chi2	97.0	79.9	11.6
	CU.KL – oli.KL	96.2	81.6	11.1
	CU.KL – signature	99.4	63.2	18.7
**Real ** ***E. coli*** ** genomes**	CU.KL – oli.chi2	89.4	81.0	14.8
	CU.KL – oli.KL	84.5	80.1	17.7
	CU.KL – signature	97.2	69.4	16.7

Our analyses also point out the particularities of each metric system. The three best tetranucleotide-based methods are those using the chi2 metric, the Kullback-Leibler divergence and the Euclidean distance. It appears that the first two are equivalent and they tend to increase dissimilarity compared to Euclidean distance. Therefore they are the metrics the most suitable for outlier detection. By using a homogenous threshold evaluation, we were able to realize a adequate comparison between the methods. This threshold determination has the advantage to be quite easy to understand and is reproducible. This is in opposition with Markov models and other classification methods that were not studied here for this reason: after a classification process one has to reasonably choose (if not) the number of groups and then identify among them the “atypical” one. Therefore, the use of metric methods using a threshold is rather straightforward. The use of a specific pre-treatment such as the “recalculation” of “a genuine recipient genome” criteria done by the signature method (see M&M) and most of all of a combination of methods allows one to tune the methods to improve the sensitivity – detect all HTs – or the specificity – do not detect native genes – of the HT detection process.

As a conclusion, we have shown that parametric methods provide a valuable tool for detecting HTs in a variety of experimental conditions. One of their advantages is that by analyzing only the DNA sequence, these methods work out partial genomes or even long stretches of DNA sequence. We demonstrated that oligonucleotide usage is a method of choice in all conditions. It was shown that the longer the oligonucleotide the better the species specificity and thus the ability to detect inclusions of foreign DNA [20]. However, for statistical reasons, the length of oligonucleotides reaches a limit and in the experimental conditions used here no oligonucleotide longer than 5 nucleotides is usable except if we choose to enlarge the window size at the expense of losing the ability to detect short and medium size HT regions.

Overall, the intrinsic genome variability would be in all cases a limit to HT detection by increasing, whatever the mode of evaluation, the threshold and so decreasing the sensitivity of any methods. We propose to combine at least two types of methods to cover all possible situations and allowing, with appropriate metrics and parameters, the best possible HTs evaluation: an oligonucleotide window based method and a gene based one working with codon usage. Even if the errors inherent to these methods are added the overall benefit is worthy. We do not discard the possibility of combining more methods but it seems important to keep in mind the cost/benefit of multiplying the methods, the only aim here being net gain in sensitivity. Moreover it is possible to use specific information related to the genome under study to improve the final result. Indeed, more and more methods using specific information such as functional annotation, chromosome position, codon usage statistical learning, comparative genomics, etc. are being developed to improve the quality of detection of HTs [24], [54], [55], [56], [57], [58], [59]. A comprehensive evaluation of such methods using comparative genomics as gold standard can be found in [59]. Though, such sophisticated methods usually require additional information that is not always available as well as complex computations that are not easily operative for one who wishes to realize a precursor investigation of it's favorite genome before further in depth analyses. This benchmark of parametric methods – that can be used quite easily – allows one to rationally choose the adequate method or combination of methods for this kind of investigations, or as a first step before combining it with specific information. For instance, in the sophisticated method using statistical learning methods over codon usage in different species [57], it might be wiser to use tetranucleotide frequencies instead of codon usage.

## Supporting Information

Figure S1 Atypicity score of the genes
(4.56 MB PDF)

Table S1 Formulas used by tested methods
(0.23 MB PDF)
