# Supplementary material for: A Benchmark of Parametric Methods for Horizontal Transfers Detection
Source: PLoS One. 2010 Apr 1;5(4):e9989. doi: 10.1371/journal.pone.0009989 (PMC2848678; doi:10.1371/journal.pone.0009989)
Supplement: Figure S1 — Atypicity score of the genes (4.56 MB PDF) [file pone.0009989.s001.pdf]

Figure S1: Atypicity score of the genes along one of the “standard” model genome for each of the 16 methods.

Each dot corresponds to the score value of a gene. The color of the dot represents the origin of the gene: native genes (*i.e. E. coli* genes) are in grey; HTs are in color according to Table 1 (shades of blue are for close genomes, shades of green for intermediary genomes and pink and orange are for far genomes, see M&M). The horizontal red line corresponds to the optimal threshold value (*i.e.* the one that minimizes mean error of detection, see M&M). (See figures on following pages)

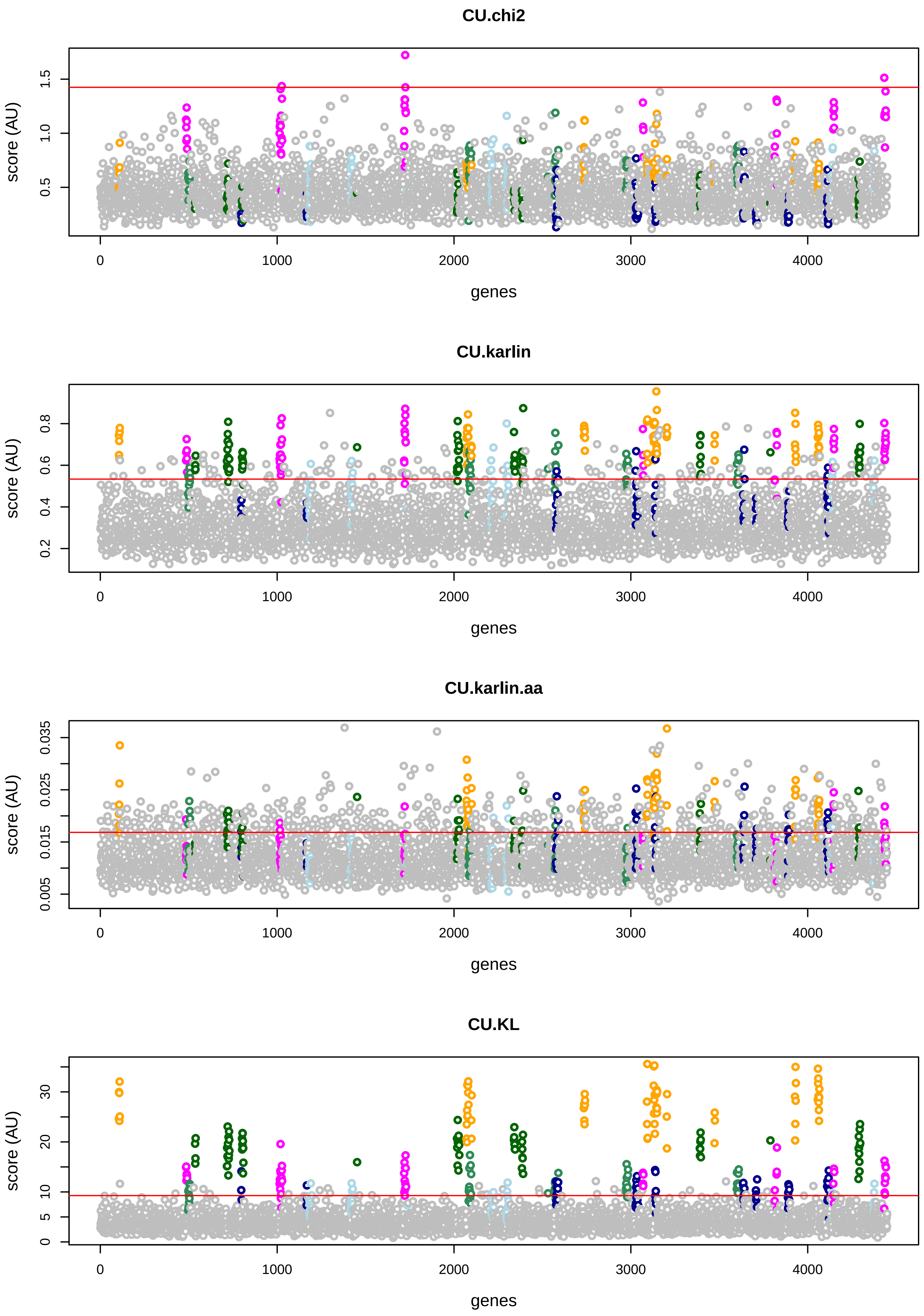
CU.chi2
score (AU)
0.5
1.0
1.5
0
1000
2000
3000
4000
genes
CU.karlin
score (AU)
0.2
0.4
0.6
0.8
0
1000
2000
3000
4000
genes
CU.karlin.aa
score (AU)
0.005
0.015
0.025
0.035
0
1000
2000
3000
4000
genes
CU.KL
score (AU)
0
5
10
20
30
0
1000
2000
3000
4000
genes

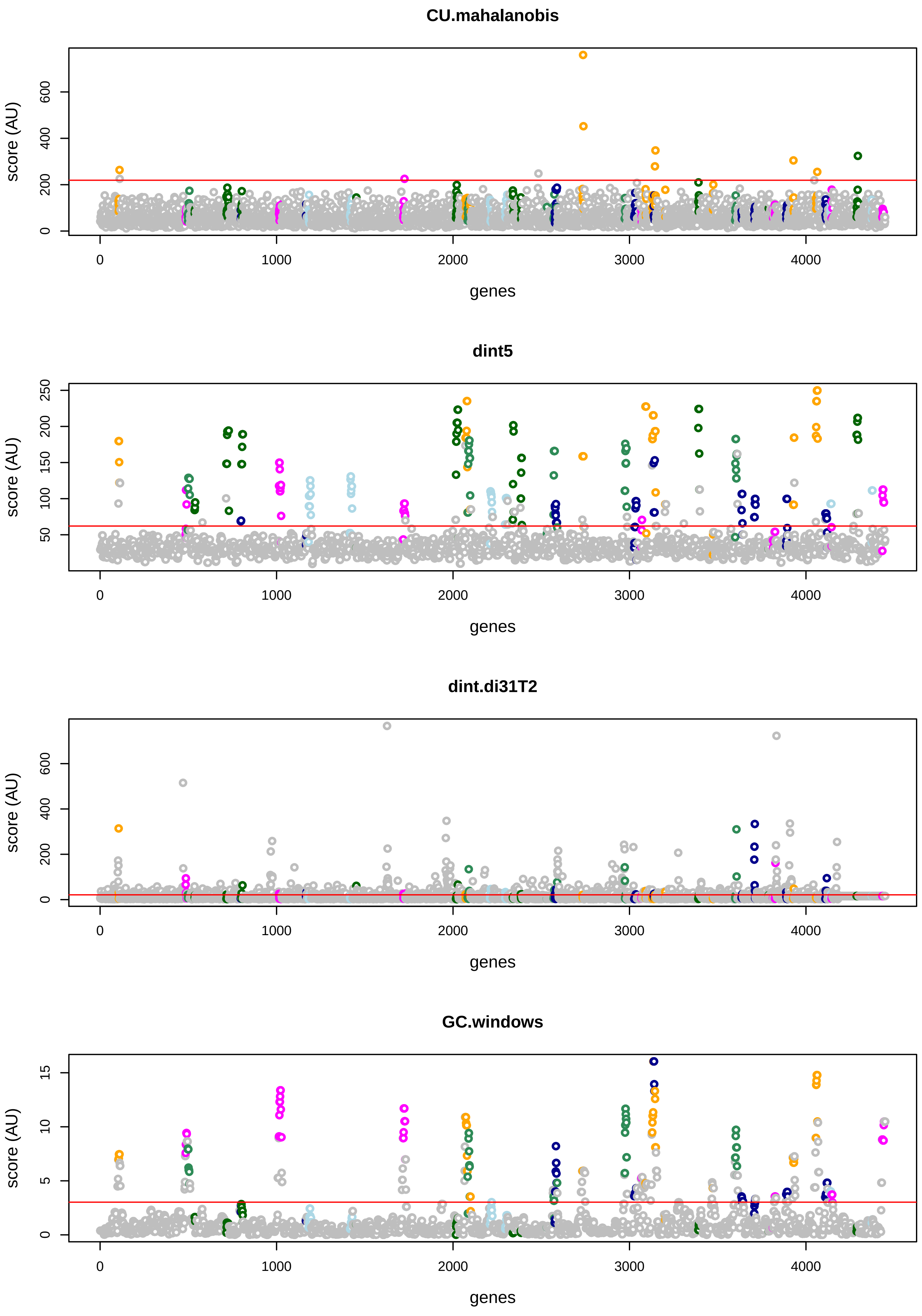
CU.mahalanobis
score (AU)
0
200
400
600
genes
0
1000
2000
3000
4000
dint5
score (AU)
50
100
150
200
250
genes
0
1000
2000
3000
4000
dint.di31T2
score (AU)
0
200
400
600
genes
0
1000
2000
3000
4000
GC.windows
score (AU)
0
5
10
15
genes
0
1000
2000
3000
4000

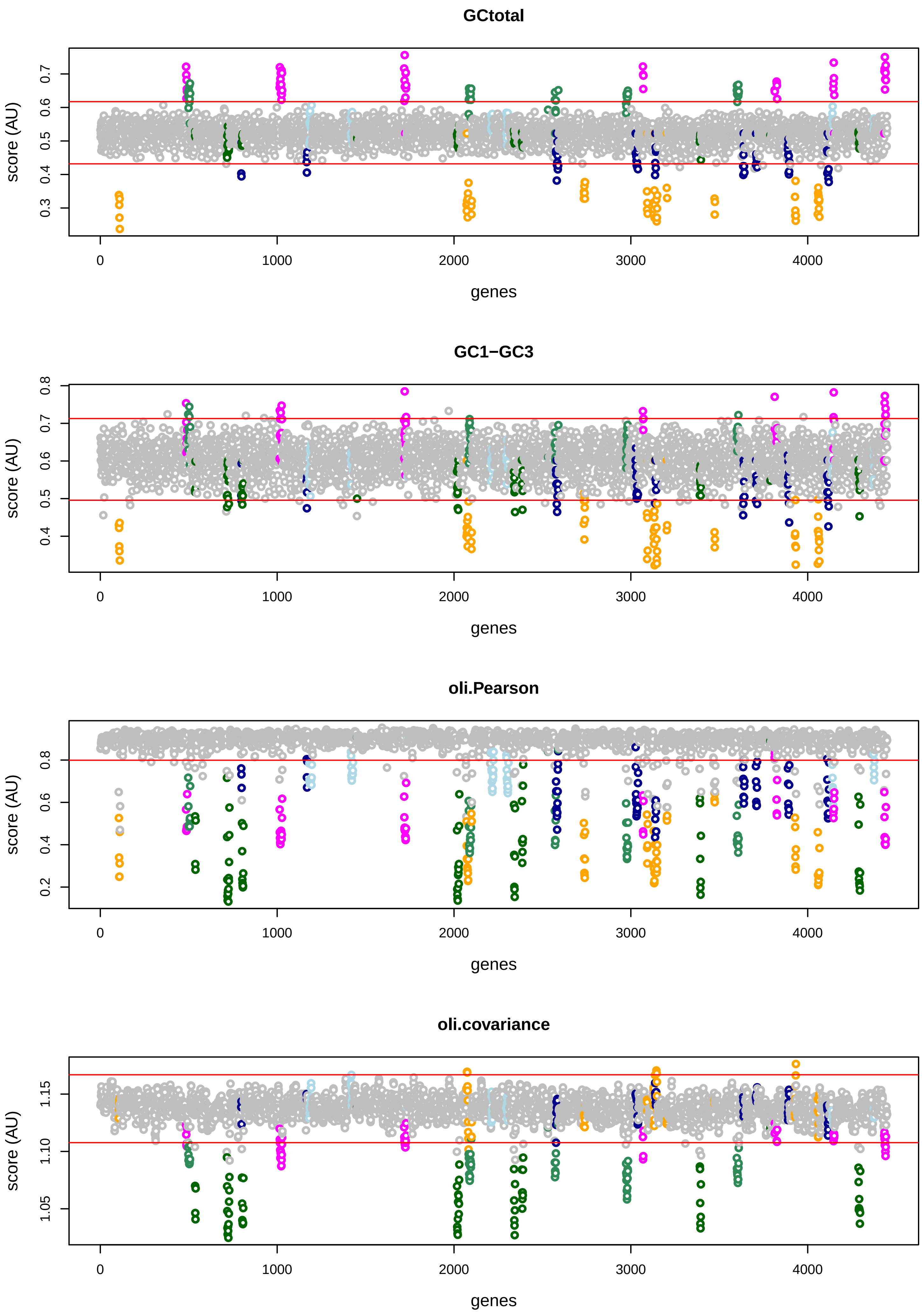
GCtotal
score (AU)
0.3
0.4
0.5
0.6
0.7
0
1000
2000
3000
4000
genes
GC1−GC3
score (AU)
0.4
0.5
0.6
0.7
0.8
0
1000
2000
3000
4000
genes
oli.Pearson
score (AU)
0.2
0.4
0.6
0.8
0
1000
2000
3000
4000
genes
oli.covariance
score (AU)
1.05
1.10
1.15
0
1000
2000
3000
4000
genes

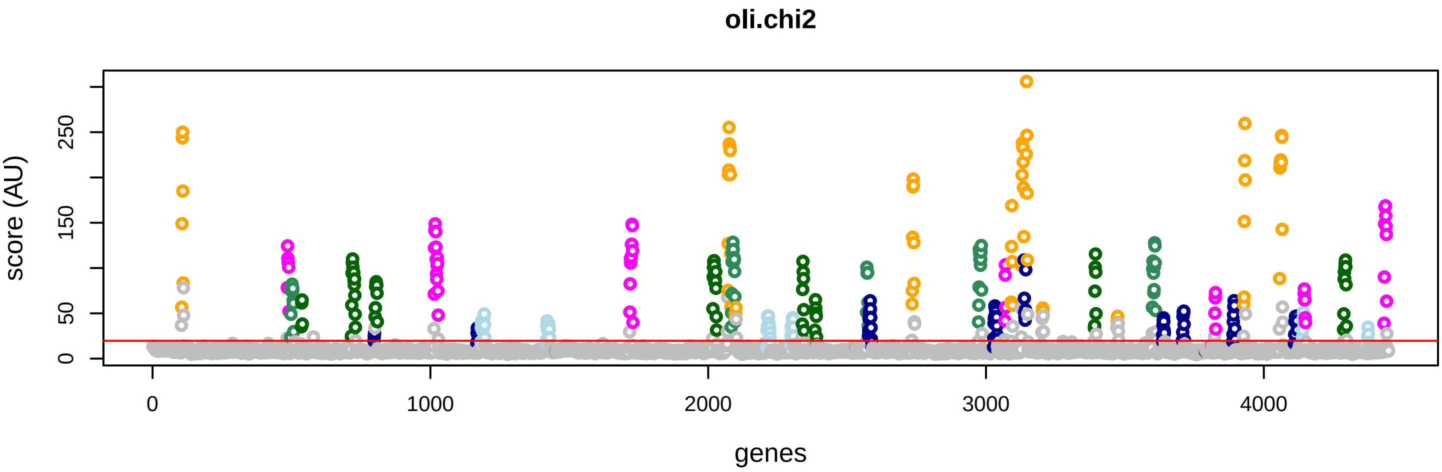
oli.chi2
score (AU)
0
50
150
250
genes
0
1000
2000
3000
4000

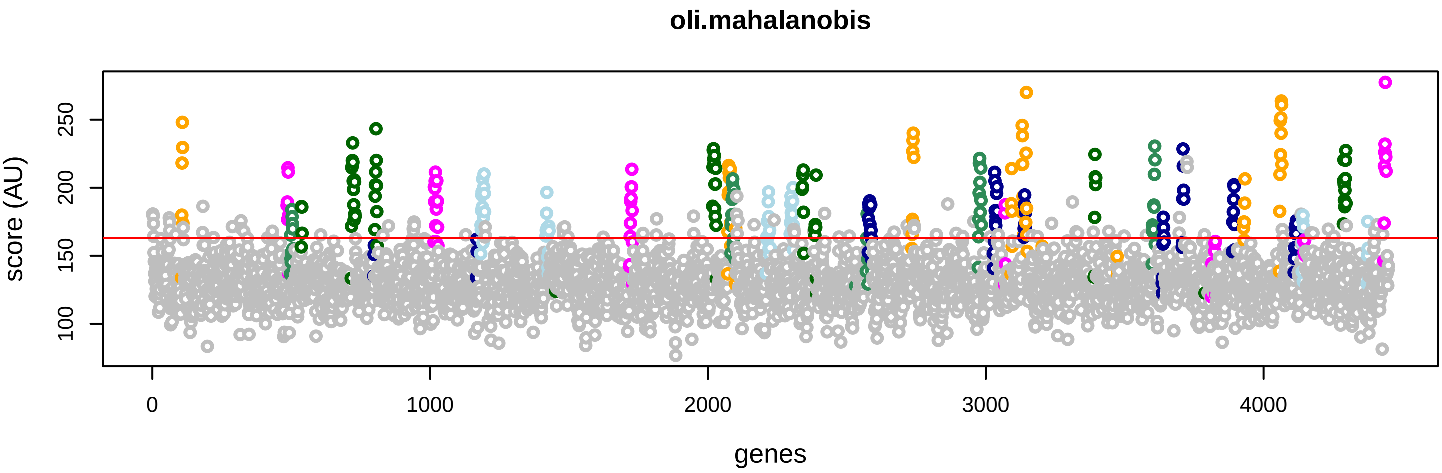
oli.mahalanobis
score (AU)
100
150
200
250
genes
0
1000
2000
3000
4000

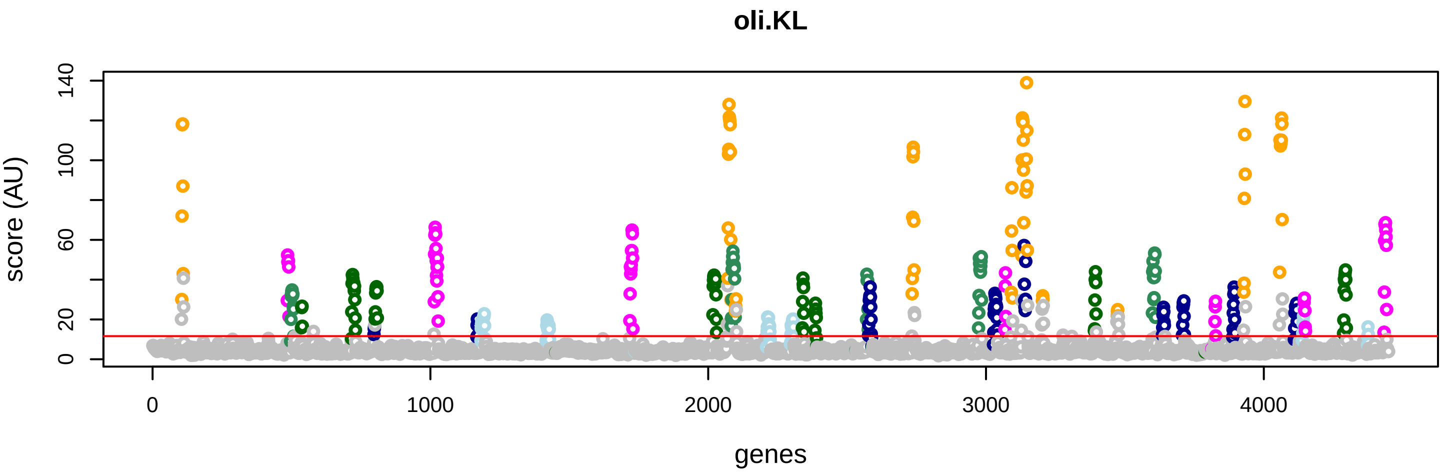
oli.KL
score (AU)
0
20
60
100
140
genes
0
1000
2000
3000
4000

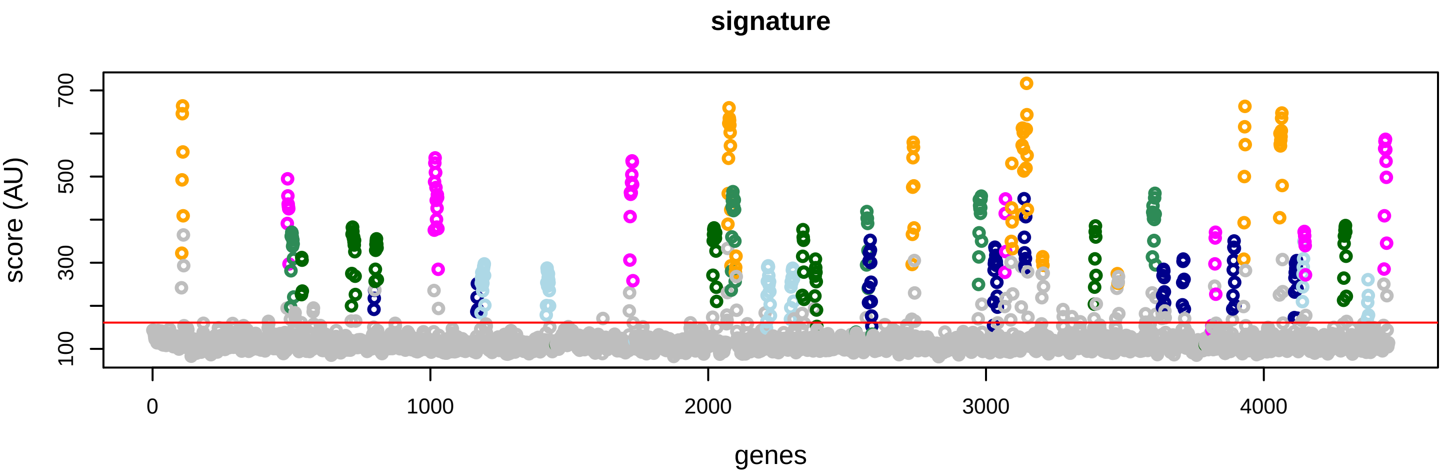
signature
score (AU)
100
300
500
700
genes
0
1000
2000
3000
4000
