# Supplementary material for: A Benchmark of Parametric Methods for Horizontal Transfers Detection
Source: PLoS One. 2010 Apr 1;5(4):e9989. doi: 10.1371/journal.pone.0009989 (PMC2848678; doi:10.1371/journal.pone.0009989)
Supplement: Table S1 — Formulas used by tested methods (0.23 MB PDF) [file pone.0009989.s002.pdf]

Table S1. Details of the metric measures used by each method.

Name of the metric measure	Formula
Manhattan	$D(g,G)=\sum_{i}^{m}\lvert f_g^i - f_G^i\rvert$
Delta*	$\delta^*(g,G)=\frac{1}{m}\sum_{i}^{m}\lvert f_g^i - f_G^i\rvert$
Euclidean	$D(g,G)=\sqrt{\sum_{i}^{m}(f_g^i - f_G^i)^2}$
Covariance	$D(g,G)=\frac{1}{m}\sum_{i}^{m} f_g^i \cdot f_G^i$
Correlation	$D(g,G)=\frac{\sum_{i}^{m}(f_g^i-\bar{f}_g)\cdot(f_G^i-\bar{f}_G)}{\sqrt{\sum_{i}^{m}(f_g^i-\bar{f}_g)^2}\cdot\sqrt{\sum_{i}^{m}(f_G^i-\bar{f}_G)^2}}$
Kullback-Leibler	$D(g,G)=\sum_{i}^{m} f_g^i \cdot \ln\frac{f_g^i}{f_G^i}$
Chi^2	$D(g,G)=\sum_{i}^{m}\frac{(f_g^i-f_G^i)^2}{f_G^i}$
Mahalanobis	$S(g,G)=(f_g-f_G)^T\cdot S^{-1}\cdot(f_g^i-f_G^i)$

D(g,G), $\delta^*(g,G)$ or *S(g,G)* is the distance/score of gene *g* to/in genome *G; m* is the number of attributes of the criterion under consideration (for example *m*=16 for dinucleotides); f_g^i is the frequency of attribute *i* for gene *g* and f_G^i it's frequency in the complete genome; $\bar{f}_g$ and $\bar{f}_G$ correspond to the mean values over all attributes calculated for gene *g* and for genome *G;* f_g corresponds to the vector of all frequencies of the criterion evaluated (for example the 16 frequencies of all dinucleotides) in gene *g* and S^{-1} corresponds to the covariance matrix.
